# Offloading Role of a Discrete Thioesterase in Type II Polyketide Biosynthesis

**DOI:** 10.1128/mBio.01334-20

**Published:** 2020-09-15

**Authors:** Kangmin Hua, Xiangyang Liu, Yuchun Zhao, Yaojie Gao, Lifeng Pan, Haoran Zhang, Zixin Deng, Ming Jiang

**Affiliations:** a State Key Laboratory of Microbial Metabolism, Joint International Research Laboratory of Metabolic & Developmental Sciences and School of Life Sciences and Biotechnology, Shanghai Jiao Tong University, Shanghai, People’s Republic of China; b State Key Laboratory of Bioorganic and Natural Products Chemistry, Center for Excellence in Molecular Synthesis, Shanghai Institute of Organic Chemistry, University of Chinese Academy of Sciences, Chinese Academy of Sciences, Shanghai, People’s Republic of China; c Department of Chemical and Biochemical Engineering, Rutgers, The State University of New Jersey, Piscataway, New Jersey, USA; John Innes Centre; McMaster University

**Keywords:** biosynthesis, natural product, offloading, thioesterase, type II polyketides

## Abstract

Type II polyketides are a group of secondary metabolites with various biological activities. In nature, biosynthesis of type II polyketides involves multiple enzymatic steps whereby key enzymes, including ketoacyl-synthase (KS_α_), chain length factor (KS_β_), and acyl carrier protein (ACP), are utilized to elongate the polyketide chain through a repetitive condensation reaction. During each condensation, the biosynthesis intermediates are covalently attached to KS_α_ or ACP via a thioester bond and are then cleaved to release an elongated polyketide chain for successive postmodification. Despite its critical role in type II polyketide biosynthesis, the enzyme and its corresponding mechanism for type II polyketide chain release through thioester bond breakage have yet to be determined. Here, kinamycin was used as a model compound to investigate the chain release step of type II polyketide biosynthesis. Using a genetic knockout strategy, we confirmed that AlpS is required for the complete biosynthesis of kinamycins. Further *in vitro* biochemical assays revealed high hydrolytic activity of AlpS toward a thioester bond in an aromatic polyketide-ACP analog, suggesting its distinct role in offloading the polyketide chain from ACP during the kinamycin biosynthesis. Finally, we successfully utilized AlpS to enhance the heterologous production of dehydrorabelomycin in Escherichia coli by nearly 25-fold, which resulted in 0.50 g/liter dehydrorabelomycin in a simple batch-mode shake flask culture. Taken together, our results provide critical knowledge to gain an insightful understanding of the chain-releasing process during type II polyketide synthesis, which, in turn, lays a solid foundation for future new applications in type II polyketide bioproduction.

## Abstract (precis)

Type II polyketides are a group of secondary metabolites with various biological activities. In nature, biosynthesis of type II polyketides involves multiple enzymatic steps whereby key enzymes, including ketoacyl-synthase (KS_α_), chain length factor (KS_β_), and acyl carrier protein (ACP), are utilized to elongate the polyketide chain through a repetitive condensation reaction. During each condensation, the biosynthesis intermediates are covalently attached to KS_α_ or ACP via a thioester bond and are then cleaved to release an elongated polyketide chain for successive postmodification.

## INTRODUCTION

Bacterial aromatic polyketides are among the most promising classes of natural products with recognized values in the pharmaceutical industry (1–3). They offer a rich molecular source for the discovery of novel therapeutic agents, including antibiotics, antifungals, antitumors, and immunosuppressants. Most known aromatic polyketides are isolated from *Actinomyces* species, a genus of Gram-positive bacteria commonly found in soil. These bacteria generally utilize the type II polyketide synthase (PKS) system to produce aromatic polyketide compounds such as daunorubicin, oxytetracycline, and kinamycins (4–7). Different from type I PKSs, which feature linearly arranged and covalently fused catalytic domains within large multimodular enzymes (8), type II PKSs are composed of dissociable enzymes (3). In a typical type II PKS system, a group of core enzymes, called the minimal PKS, encompass ketoacyl-synthase (KS_α_), chain length factor (KS_β_), and acyl carrier protein (ACP). The minimal PKS is responsible for the generation of a highly reactive poly-β-ketone backbone through a repetitive decarboxylative Claisen condensation of a malonyl-CoA extending unit with an acyl starter unit (3, 9). Following polyketide chain length maturation, mainly determined by KS_β_ (10, 11), immediate tailoring enzymes, including ketoreductases, cyclases, and aromatases, work in concert to convert a nascent reactive poly-β-keto chain into a defined polycyclic skeleton of the aromatic polyketide product (12). The thioester bond between ACP and the polyketide skeleton is then cleaved in the process called chain release, or offloading, by an unknown mechanism. The released polyketide skeleton is subsequently modified by a group of postmodification enzymes, yielding a complete molecular structure of a type II polyketide compound.

Chain release is a common and important step in the polyketide and nonribosomal peptide biosynthesis, often involving a covalent mechanism that requires an acyl-enzyme intermediate. In type I PKS and nonribosomal peptide synthetase (NRPS), thioesterase (TE)-mediated product release has been extensively investigated and well understood (13). Most TE enzymes in both type I PKS and NRPS are classified as type I TEs since they have generally been found as integrated domains at the C terminus of the assembly lines (Fig. S1). Type I TEs belong to the α/β hydrolase superfamily, which contains a conserved catalytic triad responsible for releasing product from ACP via thioester hydrolysis or O-C/N-C macrocyclization in the type I polyketide and nonribosomal peptide biosynthesis (14). In addition, many type I polyketide and nonribosomal peptide biosynthetic gene clusters (BGCs) also contain genes encoding type II TEs. Different from type I TEs that covalently attached to the terminal module of the assembly lines, type II TEs are discrete proteins. It has been shown that type II TEs possess an editing function which facilitates the removal of the aberrant acyl residues from the ACP thiol during the biosynthesis of type I polyketide and nonribosomal peptide (Fig. S1) (15, 16).

10.1128/mBio.01334-20.1 FIG S1 (A) Type I thioesterase catalyzed the termination of PKS assembly lines. (B) Type II thioesterase hydrolyzed the nonelongatable residues or incorrect start units from ACP. KS, ketosynthase; AT, acyltransferase; KR, ketoreductase; ACP, acyl carrier protein; TE, thioesterase. Download FIG S1, TIF file, 0.3 MB. Copyright © 2020 Hua et al. 2020 Hua et al. This content is distributed under the terms of the Creative Commons Attribution 4.0 International license.

On the contrary, polyketide chain release from ACP via thioester hydrolysis in the type II polyketide biosynthesis has been poorly understood. Given the fact that type II polyketide biosynthetic gene clusters often lack both integrated and discrete thioesterase genes, it is unclear whether thioester hydrolysis in the type II PKS is the enzyme-mediated or nonenzymatic reaction. It has been speculated that spontaneous hydrolysis or Aldol/Claisen condensation can facilitate the release of the aromatic polyketide skeleton from ACP (13). Yet only a few studies regarding enzyme-catalyzed type II polyketide chain release were reported so far. For example, ActIV in the actinorhodin biosynthesis pathway was characterized as a cyclase-thioesterase bifunctional enzyme which is capable of catalyzing the second-ring cyclization and the release of a bicyclic intermediate from ACP (17). In addition, the gene *grhD* in the griseorhodin biosynthetic cluster was found to encode a discrete thioesterase, suggesting its potential involvement in the polyketide chain release during the griseorhodin biosynthesis (18). However, no further characterizations have been reported to confirm this enzyme’s function. Current lack of knowledge in type II polyketide chain release calls for in-depth investigation to uncover the mechanism of this unique enzymatic reaction. To this end, we used kinamycin biosynthesis as a model system to identify and characterize the enzyme involved in the chain release.

**FIG 1:**
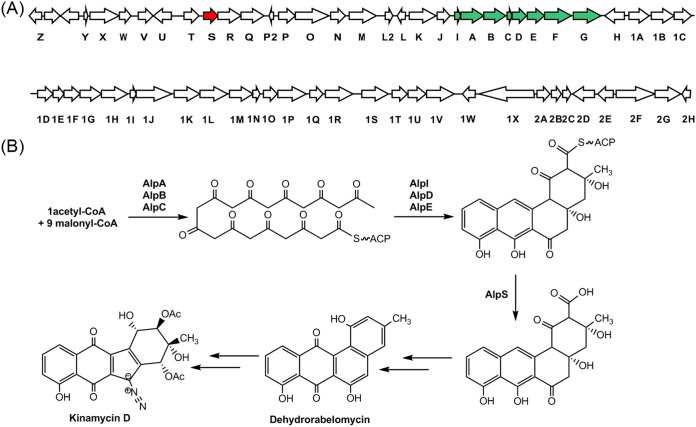
Biosynthesis of kinamycin. (A) Kinamycin biosynthetic gene cluster in Streptomyces galtieri Sgt26. The genes related with the biosynthesis of the dehydrorabelomycin are marked with green, and alpS is highlighted in red. (B) The proposed biosynthetic pathway of kinamycin.

Kinamycins belong to a small family of type II polyketides which feature a benzo[*b*]fluorene structure, a diazo functional group, and evident cytotoxic activity (6). The unique structure and potent bioactivity of kinamycin family natural products have stimulated extensive interest in understanding their biosynthesis. To this end, the kinamycin biosynthetic gene clusters from Streptomyces murayamaensis and Streptomyces ambofaciens have been cloned and sequenced (19–21). Lately, the complete kinamycin gene cluster was captured and introduced into Streptomyces albus for heterologous production (Fig. 1) (22).

On the other hand, investigations on the kinamycin biosynthesis mechanism have made great progress in recent years. The biosynthesis of the benzofluorene skeleton has been investigated, and many enzymes involved in the postmodifications have been discovered (21–24). However, the key step which hydrolyzes polyketide from ACP is still a puzzle. Here, we report the discovery and characterization of the discrete thioesterase-mediated chain release in the kinamycin biosynthesis, the first successful endeavor in type II PKS studies of this kind. A discrete thioesterase named AlpS within kinamycin BGC was first confirmed to be necessary for its biosynthesis. AlpS does not function as a typical type II thioesterase with the editing function, and both *in vivo* and *in vitro* functional characterizations suggested AlpS plays an important role in offloading the decaketide intermediate from ACP. In addition, we successfully utilized this distinct chain-releasing function of AlpS to promote the heterologous biosynthesis of the kinamycin biosynthetic intermediate, dehydrorabelomycin, in Escherichia coli, resulting in a nearly 25-fold improvement in the titer. To our knowledge, this work represents the first evidence of identification of a discrete thioesterase that directly participates in the chain-releasing step within the type II PKS biosynthesis. As such, we anticipate this discovery will facilitate better understanding of the mechanism governing type II polyketide biosynthesis as well as enable on-demand biomanufacturing of the high-value type II PK compounds.

## RESULTS

### *In vivo* analysis revealed AlpS is essential for the kinamycin biosynthesis.

Recent studies have collectively identified the complete biosynthetic cluster responsible for kinamycin and demonstrated multiple enzymatic steps in the kinamycin biosynthesis process. Accordingly, a potential biosynthetic pathway has been proposed, and the functions of many genes in the cluster have been confirmed (Fig. 1). To date, however, the specific functions of several other genes in the cluster are still not known, and many of them are thought to be involved in the generation of a diazo functional group (23). Using nucleotide sequence alignment, we found that, among the unknown genes, *alpS* encoded a unique enzyme that belongs to the α/β hydrolase family, whose members are commonly found in the type I PKSs but rarely in the type II PKS systems. Analyzing the organization of the kinamycin’s BGC reveals that *alpS* may be transcriptionally connected with two neighboring genes, *alpR* and *alpQ*, that code for one pair of KS_α_ and KS_β_. Previous work has shown that *alpR* and *alpQ* are not directly involved in the biosynthesis of kinamycin (20).

**FIG 2:**
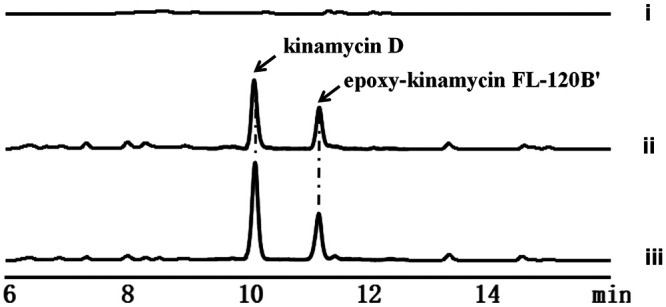
HPLC analysis of the knockout and complement experiments of *alpS* in *S. albus* J1074. (i) S. albus J1074 (2E9 Δ*alpW* Δ*alpS*); (ii) S. albus J1074 (2E9 Δ*alpW* Δ*alpS*::*alpS*); (iii) S. albus J1074 (2E9 Δ*alpW*) (control). HPLC traces were recorded at 424 nm.

To investigate the importance of AlpS in the biosynthesis of kinamycin, a 780-bp internal fragment of *alpS* gene was in-frame deleted in the bacterial artificial chromosome (BAC) clone 2E9 Δ*alpW* containing the entire kinamycin BGC (22). The resulting BAC 2E9 Δ*alpW* Δ*alpS* strain was transferred into S. albus by intergenic conjugation with E. coli. The Δ*alpS* transformant was verified by PCR amplification. Analysis of the culture of the Δ*alpS* strain revealed no detectable level of kinamycin even after 48 h cultivation, indicating that the kinamycin biosynthesis was abolished in this knockout strain (Fig. 2). Furthermore, no other related metabolites were found to be accumulated in the Δ*alpS* strain. To exclude any possible polar effect, the *alpS* deletion mutant was complemented with a single copy of *alpS*, which was placed downstream of the *kasO*p* promoter through intergeneric conjugation. High-pressure liquid chromatography (HPLC) analysis showed the production of kinamycin of the complemented strain was restored to a similar level to that of the wild-type strain. Collectively, these results unambiguously confirmed the essential role of the AlpS for the biosynthesis of kinamycin.

### *In vitro* functional assay verified a thioesterase activity of AlpS.

**FIG 3:**
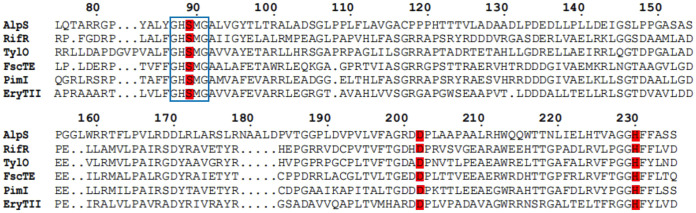
Sequence alignment of AlpS and other type II TEs with the editing function in type I PKSs. The conserved GHSMG motifs are marked by rectangles, and the Ser-His-Asp triad is highlighted in red.

**FIG 4:**
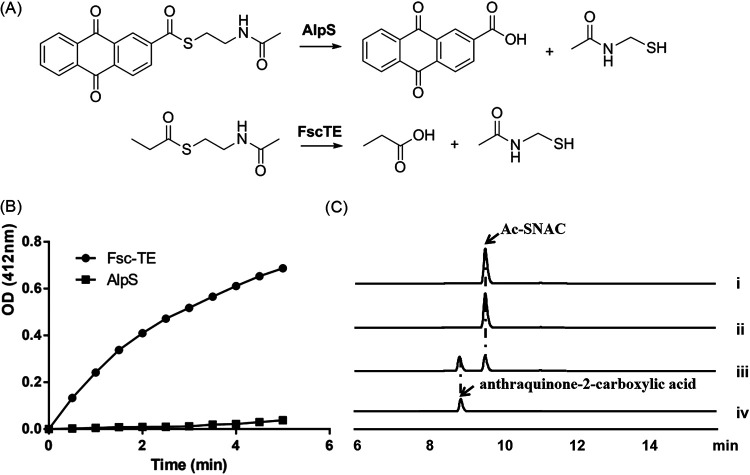
Analysis of the hydrolytic activity of AlpS toward SNAC esters. (A) Hydrolysis of Ac-SNAC by AlpS and propionyl-SNAC by FscTE. (B) Time courses of AlpS (■) and Fsc-TE (●) hydrolysis of propionyl-SNAC. (C) HPLC analysis of hydrolysis Ac-SNAC: (i) Ac-SNAC standard; (ii) Ac-SNAC with boiled AlpS; (iii) Ac-SNAC with AlpS; (iv) anthraquinone-2-carboxylic acid standard. HPLC traces were recorded at 276 nm.

Upon inspecting the amino acid sequence of AlpS, we discovered a GHSMG motif that is highly conserved in the type II thioesterases with the editing function (Fig. 3) (25). Further bioinformatic analysis revealed that AlpS belongs to the InterPro family IPR012223, indicating its potential role in the editing process during kinamycin biosynthesis. In addition, an HHpred structure homologue search showed that AlpS has a high similarity to RifR, a well-known type II thioesterase with an editing function in the rifamycin biosynthesis (26). It should be noted that, to date, only a few type II polyketide BGCs have been found to contain genes encoding type II thioesterase, most of which influence the production via their editing or starter unit selection functions. To uncover the putative function of AlpS, its variant with the hexahistidine tag fused at the N terminus was overexpressed in E. coli and purified to homogeneity by affinity chromatography (Fig. S2). Hydrolytic activity of purified AlpS was then investigated using the *N*-acetylcysteamine thioester propionic acid (hereafter termed propionyl-SNAC), a substrate mimic of the short-chain acyl thioester. Since propionyl-SNAC is structurally distinct from the native intermediates of the kinamycin biosynthesis, the ability to hydrolyze propionyl-SNAC would infer that the AlpS has the editing function, akin to a removal process of the aberrant acyl moiety from ACP. Using the previously reported dithionitrobenzoic acid (DTNB)-based spectrophotometric assay, we observed only a minimal hydrolytic activity of AlpS toward propionyl-SNAC (Fig. 4B). On the contrary, over 85% of the propionyl-SNAC was hydrolyzed within 5 min under the same reaction condition when using a positive-control enzyme FscTE, a well-known type II thioesterase with reported editing function (27). A similar phenomenon was observed when butyryl-SNAC was used as a substrate (Fig. S3). These results strongly suggest that AlpS does not function as an editing enzyme in the kinamycin biosynthesis.

10.1128/mBio.01334-20.2 FIG S2 Purification of AlpS analyzed by SDS-PAGE. Lane 1, purified AlpS; lane 2, protein molecular weight marker; lane 3, whole-cell lysate of induced cells carrying pET28a *alpS*; lane 4, soluble fraction from induced cells carrying pET28a *alpS*. Download FIG S2, TIF file, 2.0 MB. Copyright © 2020 Hua et al. 2020 Hua et al. This content is distributed under the terms of the Creative Commons Attribution 4.0 International license.

10.1128/mBio.01334-20.3 FIG S3 Time courses of AlpS (■) and Fsc-TE (●) hydrolysis of butyryl-SNAC. Download FIG S3, TIF file, 0.1 MB. Copyright © 2020 Hua et al. 2020 Hua et al. This content is distributed under the terms of the Creative Commons Attribution 4.0 International license.

Encouraged by these results, we sought to establish that AlpS represents the first novel group of thioesterase involved in the chain release of polyketide chain from ACP during the type II polyketide biosynthesis process. To this end, a potential offloading activity of AlpS was tested using anthraquinone-2-carboxylic acid-*N*-acetylcysteamine (hereafter termed Ac-SNAC) substrate, which is widely used as an aromatic polyketide ACP thioester analog (Fig. 4A) (28). Upon incubating the purified AlpS with Ac-SNAC, a production of the hydrolytic product, anthraquinone-2-carboxylic acid, was clearly detected, and about half of the hydrolysis of Ac-SNAC was observed within 30 min according to the HPLC analysis (Fig. 4C). In comparison, no detectable hydrolytic product was observed in a control reaction where purified AlpS was replaced with a heat-inactivated version of AlpS. It is therefore clearly indicated that AlpS can indeed catalyze the hydrolysis of the thioester bond in Ac-SNAC. Given that AlpS is required for the kinamycin biosynthesis and it has a strong hydrolytic activity toward Ac-SNAC, we speculated that the function of AlpS is to hydrolyze the acyl-ACP thioester bond and thus facilitate the release of the decaketide intermediate from ACP (AlpC) during the kinamycin biosynthesis.

**FIG 5:**
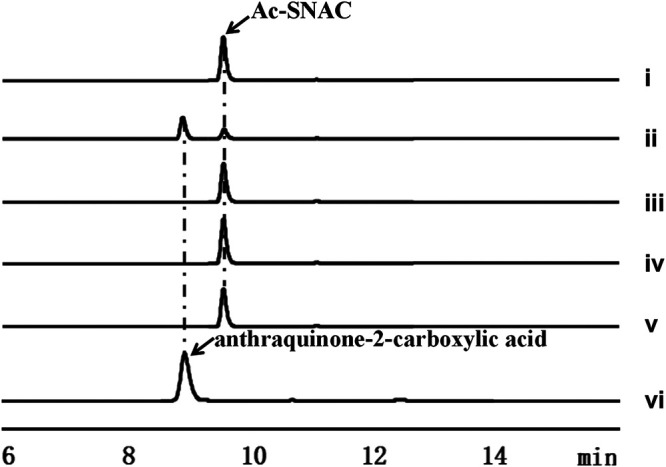
AlpS hydrolysis activity toward Ac-SNAC is dependent on the catalytic triad. HPLC analysis of the hydrolytic activity of AlpS and its catalytic triad mutants. (i) Ac-SNAC standard; (ii) Ac-SNAC with AlpS; (iii) Ac-SNAC with AlpS S89A mutant; (iv) Ac-SNAC with AlpS D202N mutant; (v) Ac-SNAC with AlpS H230A mutant; (vi) anthraquinone-2-carboxylic acid standard. HPLC traces were recorded at 276 nm.

A multiple-sequence alignment also reveals a conserved catalytic triad at the Ser89, Asp202, and His230 residues of AlpS (Fig. 3). Since the catalytic triad is a conserved motif commonly found within the member of the α/β hydrolase family, we hypothesized that these residues are crucial for the hydrolytic activity of the AlpS. To test this, we made three AlpS mutants where Ser89, Asp202, and His230 residues were substituted with Ala, Asn, and Ala, respectively. Hydrolytic activity toward the Ac-SNAC substrate of these mutants was then tested in comparison with the wild-type enzyme. Following an *in vitro* hydrolysis assay, the reaction mixture was analyzed using HPLC (Fig. 5). As anticipated, incubating Ac-SNAC with a wild-type AlpS enzyme for 1 h led to efficient hydrolysis of the substrate, as evidenced by the clear appearance of a major peak corresponding to the hydrolyzed product (around 75%) (Fig. 5ii). In comparison, substituting the wild-type AlpS with its mutants completely abolished the hydrolysis of the Ac-SNAC (Fig. 5iii to Fig. 5v). It is therefore demonstrated that all three amino acid residues within the catalytic triad are necessary for the hydrolytic function of AlpS. To further confirm the role of the AlpS catalytic triad in the kinamycin biosynthesis, we placed S89A mutant *alpS* under the control of the *kasO*p* promoter to complement the *alpS* deletion strain through intergeneric conjugation. Consistent with our *in vitro* activity data, the S89A mutant *alpS* could not restore the production of kinamycin in the *alpS* deletion strain, indicating that the catalytic triad is critical for AlpS’s *in vivo* function (data not shown). Together, our results for manipulating the conserved triad region hereby further indicated that AlpS has the thioesterase activity for offloading a polyketide chain during kinamycin biosynthesis.

### Phylogenic studies of AlpS and its homologues.

**FIG 6:**
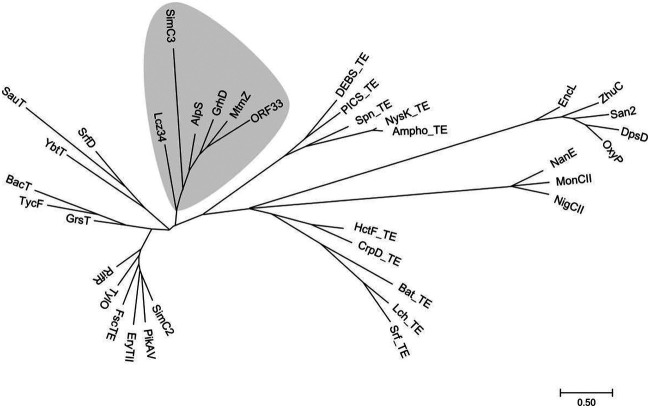
The phylogenetic of the characterized TE proteins (domains) and some discrete thioesterases in type II polyketide biosynthetic clusters. AlpS, MtmZ, X26-TEII (ORF33), GrhD, Lcz34, San2, SimC2, SimC3, SauT, EncL, ZhuC, DpsD, and OxyP are discrete thioesterases found in type II polyketide biosynthetic clusters. TylO, FscTE, RifR, PikAV, EryTII, MonCII, NanE, and NigCII are type II TEs in type I PKSs. GrsT, TycF, BacT, SrfD, and YbtT are type II TEs in NRPSs. DEBS_TE, PICS_TE, NysK_TE, Ampho_TE, and Spn_TE are type I TE domains in type I PKSs. Lch_TE, Bat_TE, Srf_TE, HctF_TE, and CrpD_TE are type I TE domains in NRPSs. The accession numbers of the proteins used in this phylogenetic tree are listed in Table S3.

Since no thioesterase involved in the type II polyketide chain release has been reported previously, the identification of a discrete thioesterase, AlpS, as an offloading enzyme brings up the possibility that there are more similar enzymes in other type II polyketide BGCs. As AlpS contains a GHSMG motif that is highly conserved in type II thioesterases with editing functions, it is of great interest to investigate the phylogenic relationship of AlpS with other well-characterized thioesterases, such as the type II thioesterase from polyketide and NRP biosynthesis and the type I thioesterase domains in modular PKS and NRPS. With AlpS as a new member of the thioesterase family involved in natural product biosynthesis, we searched the NCBI database to find more AlpS-like enzymes. This effort resulted in the identification of additional 12 discrete thioesterases in the type II polyketide BGCs, including EncL, which functions as an editing enzyme to remove aberrant starter units (29). To demonstrate a genetic hierarchy of these thioesterases, we constructed a phylogenetic tree using MEGA7 with a maximum likelihood method (Fig. 6). This phylogenetic tree clearly demonstrated the relationship between PKS type II TEs, NRPS type II TEs, PKS type I TE domains, NRPS type I TE domains, and their homologues, respectively. Notably, AlpS and the 5 other thioesterases from the type II polyketide BGCs form a unique clade, indicating that they may have similar chain-releasing functions in their own biosynthetic pathways. The other thioesterases in type II polyketide biosynthesis that have editing functions form another distinct clade separated from the AlpS clade. Nonetheless, further identification of AlpS-like enzymes associated with the type II polyketide biosynthesis will facilitate better understanding of the underlying mechanism governing the offloading process during the biosynthesis of this family of natural products.

### Enhancing dehydrorabelomycin production yield in E. coli via the introduction of the *alpS* gene.

**FIG 7:**
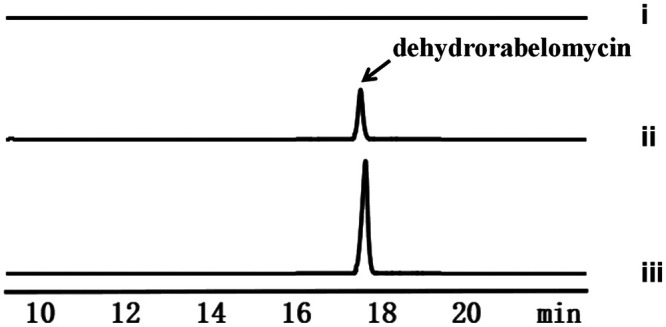
The influence of AlpS in the E. coli dehydrorabelomycin production. (i) E. coli BAP1/pGro7/pXY-2/pXY-3/pXY-6 without IPTG inducing (control); (ii) E. coli BAP1/pGro7/pXY-2/pXY-3/pXY-6; (iii) E. coli BAP1/pGro7/pKM-1/pXY-3/pXY-6 (the crude extract was diluted 10 times before HPLC analysis). pKM-1contains one copy of *alpS*. HPLC traces were recorded at 424 nm.

**FIG 8:**
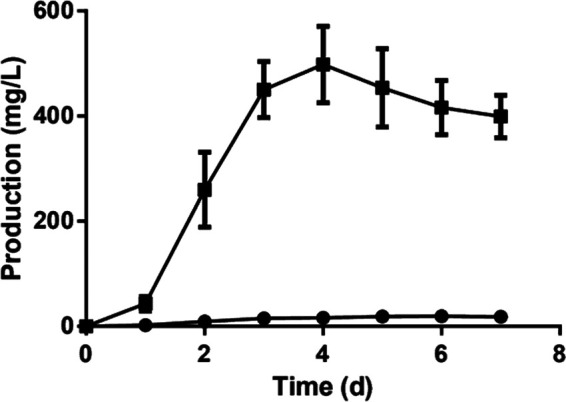
Time courses of the dehydrorabelomycin production by BAP1/pGro7/pKM-1/pXY-3/pXY-6 (■) and BAP1/pGro7/pXY-2/pXY-3/pXY-6 (●). The error bars represent standard deviation of the experimental measurements for three independent experiments.

Recently, we reconstituted the biosynthesis of dehydrorabelomycin, a key precursor of kinamycin and various other compounds from the angucycline family, in a heterologous host E. coli (30). In that study, engineered E. coli strain yielded ∼20 mg of dehydrorabelomycin per liter of culture within 7 days, even in the absence of the *alpS* gene in the engineered biosynthetic pathway. We reasoned that certain E. coli endogenous enzymes could complement, at least partially, the hydrolysis function of AlpS. The discovery of the AlpS function in this work motivated us to introduce AlpS into our orthogonal biosynthetic pathway in E. coli to further enhance the dehydrorabelomycin production yield. Toward this end, AlpS was overexpressed under a strong T7 promoter in the previous dehydrorabelomycin-producing strain. As expected, the engineered strain overexpressing AlpS generated a significant increase in the production of dehydrorabelomycin. In fact, this overproduction led to an easily observed colorimetric change, from pale yellow to black, 24 h after the gene expression induction. HPLC analysis of the extracted product from the AlpS overexpressed strain at day 4 showed an over 25-fold increase in the dehydrorabelomycin production compared to the nonoverexpressed control (Fig. 7). Furthermore, a time course analysis over 7 days of the dehydrorabelomycin production in the strains with and without AlpS overexpression was also conducted. As shown in Fig. 8, when AlpS was overexpressed in E. coli, dehydrorabelomycin production was around 30 times faster than the control strain in the first 3 days, and the production level reached its saturation at around day 4. The final dehydrorabelomycin production in a simple 250-ml shake flask culture was 0.50 g/liter at day 4. Without the use of advanced bioreactor techniques, this biosynthetic yield is already outstanding compared to all other studies for heterologous biosynthesis of polyketide natural product in E. coli. Overall, such significant improvement in the heterologous dehydrorabelomycin production suggests that AlpS’s thioesterase activity indeed facilitates the efficient offloading of the decaketide intermediate from PKS, which, in turn, increases the turnover rate of the PKS for new biosynthesis cycles and improves the biosynthesis performance.

## DISCUSSION

Polyketides are among the largest family of natural products with large structural diversity as well as numerous valuable bioactivities. Type I polyketide synthases are multifunctional enzymes composed of functional modules, each of which catalyzes one cycle of the polyketide chain elongation and, subsequently, the associated modification. After a polyketide chain has matured in its length, the chain is then released from the ACP in the process catalyzed by the TE domains usually located at the C terminus of PKSs. Some of the type I PKSs responsible for the biosynthesis of polyether ionophores, such as nachangmycin (31) and monensin (32), however, contain no TE domain at the terminus of the PKSs but, rather, utilize specific discrete thioesterase, such as NanE in the nachangmycin biosynthesis, to release the polyether chains from the assembly line. It is worth noting that these polyether TEs are phylogenetically different from both the editing type II TE and the integrated type I TE.

On the other hand, many type II polyketide BGCs do not contain genes encoding discrete thioesterases, and only a few type II thioesterases with editing functions have been identified to date, including the ZhuC enzyme from R1128 PKS (33) and the EncL enzyme from the enterocin PKS (29). These enzymes have been found in some type II polyketide biosynthetic pathways that utilize nonacetate starters. For example, ZhuC was originally proposed as a malonyl-CoA:ACP transferase dedicated for the initiation module (34) but later has been determined to be a thioesterase with an editing function to remove acetate starter units that will otherwise compete with the alkyl-ZhuG species for priming the elongation PKS module (33).

While the product offloading in both type I polyketide and nonribosomal peptide biosynthesis have been well investigated, the release of the type II polyketide chain remains poorly understood. Recent studies in the actinorhodin biosynthesis demonstrated that ActIV is a bifunctional enzyme that could catalyze both the formation of the second polyketide ring and the release of the polyketide chain from ACP. However, to our knowledge, no discrete thioesterase has been found to be responsible for the release of type II polyketide from ACP.

In this study, we demonstrated that AlpS is essential for the kinamycin biosynthesis. The inability to produce kinamycin in the Δ*alpS* mutant strain indicated that AlpS does not function as a regular type II thioesterase to remove undesirable substrates because it would otherwise alter the product profile and decrease the final production yield, rather than completely abolish the biosynthesis. Consistent with this result, the *in vitro* experiment proved that AlpS does not efficiently hydrolyze propionyl-SNAC, confirming its lack of proofreading activity for hydrolyzing the incorrect substrates. Furthermore, the high hydrolytic activity of AlpS toward a thioester bond within the aromatic polyketide-ACP thioester analog, Ac-SNAC, suggested that it plays a critical role in a chain-releasing step via thioesterase bond breakage. Subsequent catalytic triad mutation and heterologous biosynthesis enhancement experiments further support our hypothesis on the AlpS’s chain-releasing function. To the best of our knowledge, this is the first report of a dedicated discrete thioesterase enzyme characterized as a chain-releasing enzyme for the type II polyketide biosynthesis. As the information on the chain release mechanism of the cyclized acyl-*S*-ACP in the type II polyketide biosynthesis has been limited so far, we anticipate that this work will lay a solid foundation for further investigation of this historically mysterious group of enzymes, which, in turn, will help to broaden our knowledge about type II polyketide biosynthesis. To this end, further structural characterization may help us better understand the substrate specificity of AlpS as an offloading enzyme in type II polyketide biosynthesis. Moreover, a better understanding of the function and mechanism of AlpS can facilitate future efforts in enhancing type II polyketide bioproduction as well as in engineering biosynthesis of the type II polyketide to produce novel bioactive derivatives or analogs.

## MATERIALS AND METHODS

### General materials and procedures.

All chemicals and reagents were purchased from commercial sources without further purification. DMAP (4-dimethylaminopyridine) was purchased from Solarbio (Beijing, China). *N*-(3-dimethylaminopropyl)-*N*′-ethylcarbodiimide hydrochloride (EDC-HCl) was purchased from AnaSpec (USA), and NAC (*N*-acetylcysteamine) was from Santa Cruz Biotechnology (USA). Anthraquinone-2-carboxylic acid was purchased from TargetMol (USA). One liter of production medium contained 10 g tryptone, 10 g sodium chloride, 5 g yeast extract, 15 g glycerol, and 100 mM HEPES buffer and was adjusted to pH 7.6 before use. All the strains and plasmids used in this work are listed in Table S1, and primers are listed in Table S2. The E. coli strain BAP1 was provided by Blaine Pfeifer at The State University of New York at Buffalo and fermented in production medium at 18°C. E. coli strains were cultured in LB media at 37°C; S. albus J1074 and the derived strains were cultured on Mannitol soya flour (MS) plates for sporulation and conjugation at 30°C. The fermentation of *Streptomyces* spp. was carried out in 30 ml R2 liquid medium containing 1.5 g HP-20 resin at 30°C.

10.1128/mBio.01334-20.5 TABLE S1 Strains and plasmids used in this study. Download Table S1, DOCX file, 0.01 MB. Copyright © 2020 Hua et al. 2020 Hua et al. This content is distributed under the terms of the Creative Commons Attribution 4.0 International license.

10.1128/mBio.01334-20.6 TABLE S2 Primers used in this study. Download Table S2, DOC file, 0.02 MB. Copyright © 2020 Hua et al. 2020 Hua et al. This content is distributed under the terms of the Creative Commons Attribution 4.0 International license.

10.1128/mBio.01334-20.7 TABLE S3 Protein IDs of the TEs used in the phylogenetic analysis. Download Table S3, DOC file, 0.04 MB. Copyright © 2020 Hua et al. 2020 Hua et al. This content is distributed under the terms of the Creative Commons Attribution 4.0 International license.

### Inactivation of *alpS* and its complementation.

The *alpS* deletion experiment was conducted on the BAC clone 2E9 Δ*alpW* using the PCR targeting technique. The amplified gene deletion cassette was generated through PCR amplification of the kanamycin-resistant gene (*neo*). The PCR product was purified and introduced into E. coli BW25113/pIJ790/2E9 Δ*alpW* by electroporation to replace *alpS* in 2E9. The *alpS* deletion construct was confirmed by PCR, and the in-frame deletion construct was obtained by FLP-mediated excision to remove FLP recombination target (FRT)-*neo*-FRT cassette. The resulting final *alpS* deletion construct was PCR verified and introduced into S. albus J1074 using E. coli ET12567/pUB307-mediated triparental conjugation. The *alpS* gene was PCR amplified from BAC 2E9, digested by NdeI/EcoRI, and ligated to the NdeI/EcoRI-digested pJTU968 *kasO*p*. Then, the *kasO*p* *alpS* fragment was obtained through MunI/EcoRI digestion of pJTU968 *kasO*p* *alpS* and ligated to pPM927 (EcoRI single digested and dephosphorylated) to generate pPM927 *kasO*p* *alpS*. The resulting pPM927 *kasO*p* *alpS* was introduced into the S. albus J1074 (2E9 Δ*alpW* Δ*alpS*) to obtain the complementation strain S. albus J1074 (2E9 Δ*alpW* Δ*alpS*)*/*pPM927 *kasO*p* *alpS.*

### Fermentation and HPLC analysis.

*Streptomyces* spp. were grown on MS plates at 30°C for sporulation, and then the spores were transferred to 30 ml tryptic soy broth supplemented with 0.5% yeast extract (TSBY) liquid medium in a triangular flask and incubated with shaking at 30°C for 1 day as a seed culture. Then, 1 ml seed culture was added to 30 ml R2 liquid medium with 1.5 g HP-20 resin in a triangular flask and fermented at 30°C for 2 to 3 days. After fermentation, 300 μl (1%) glacial acetic acid was added to the 30-ml culture. The culture were then extracted by equal volumes of ethyl acetate twice. The organic fractions were combined and dried by rotary evaporation. The dried residue was dissolved in 600 μl methanol.

For heterologous production of dehydrorabelomycin, E. coli BAP1/pGro7/pXY-2/pXY-3/pXY-6 was used as the starting strain for the strain construction of this work (30). The gene *alpS* was inserted into pXY-2 just behind MCAT (malonyl-CoA: acyl carrier protein transacylase) to form pKM-1 by HindIII and SpeI digestion and isocaudarner ligation as described in a previous paper (30). For fermentation, the constructed E. coli strains were inoculated into 3 ml LB liquid medium with necessary antibiotics and cultured overnight at 37°C in the shaker. The overnight culture was then diluted 1:100 to 50 ml production medium with appropriate antibiotics and grew at 37°C with shaking until the optical density at 600 nm (OD_600_) was nearly equal to 0.5. After that, isopropyl-β-d-thiogalactopyranoside (IPTG) (0.5 mM) and arabinose (3 mM) were added to induce protein expression. The fermentation was carried out at 18°C with shaking at 220 rpm for 4 days. The culture was harvested and extracted with equal volumes of ethyl acetate twice after the pH was adjusted by adding 1% glacial acetic acid. The organic phase was combined and dried by rotary evaporation. The final dried extract was dissolved in 2.4 ml dimethyl sulfoxide (DMSO).

For the time course analysis of the production using BAP1/pGro7/pKM-1/pXY-3/pXY-6 and BAP1/pGro7/pXY-2/pXY-3/pXY-6, the culture condition was the same as indicated above. We took 650 μl broth from the culture every day, and we added 6.5 μl glacial acetic acid before it was extracted with equal volumes of ethyl acetate twice. After rotary evaporation, the final extract was dissolved in 100 μl DMSO.

HPLC analysis was performed on an Agilent 1200 HPLC system using a Zorbax XDB-C18 column (5 mm, 4.6 by 250 mm). In each experiment, the following conditions were used: 75% solvent A (water with 0.1% trifluoroacetic acid [TFA]) to 100% solvent B (acetonitrile with 0.1% TFA) over 20 min and 100% solvent B for 10 min at a flow rate of 1 ml/min. The absorbance was monitored at 276 nm for Ac-SNAC hydrolysis experiments and 424 nm for kinamycin and dehydrorabelomycin detection.

### Sequence and phylogenetic analysis.

Multiple-sequence alignment was performed using ClustalW, and the figure was drawn on the website of ENDscript/ESPript. The phylogenetic tree was constructed using MEGA7 with the method of maximum likelihood, and the proteins used in this phylogenetic analysis are listed in Table S3.

### Protein expression and purification.

Proteins used in this study were all expressed in pET28a with a His_6_ tag. The plasmid was transformed to E. coli BL21(DE3)/pGro7, and the expressing strains were inoculated into 3 ml LB liquid medium with requisite antibiotics and cultured overnight at 37°C in the shaker. The overnight culture was then diluted 1:100 to 200 ml LB with appropriate antibiotics, grown at 37°C to an OD_600_ nearly equal to 0.5, and then induced for protein overexpression at 16°C for 20 h by adding IPTG (0.1 mM) and arabinose (3 mM). The cells were harvested by centrifugation at 4°C and resuspended in 50 mM Tris (pH 8.0) containing 500 mM NaCl and 10% vol/vol glycerol. Then, the cells were disrupted by sonication and purified through a nickel affinity chromatography column. The purified proteins from the affinity column were desalted by the PD10 column and concentrated by Amicon Ultra-15 centrifugal filter unit (10 kDa). The protein concentration was determined by Bradford protein assay kit (Tiangen Biotech Company).

### Preparation of NAC esters.

For the synthesis of Ac-SNAC, anthraquinone-2-carboxylic acid (20 mg, 0.05 mmol), EDC-HCl (200 mg, 1.26 mmol), and DMAP (25 mg, 0.2 mmol) were first dissolved in CH_2_Cl_2_ (10 ml). Then, *N*-acetylcysteamine (50 mg, 0.42 mmol) was added, followed by stirring at room temperature for 30 h. Then, ethyl acetate (10 ml) and 1 M hydrochloric acid (10 ml) were added to the system, followed by stirring for 10 min. After that, the aqueous and organic phases were separated by centrifugation. The aqueous phase was extracted with ethyl acetate (5 ml, 3 times). Then the organic phase was combined and washed with saturated sodium bicarbonate solution (10 ml, 2 times) and saturated sodium chloride solution (15 ml, 2 times). After all these steps, the solvent was evaporated by rotary evaporation. The crude residues were further purified by silica column chromatography (chloroform:methanol ratio of 9:1).

The synthesis of propionyl-SNAC and butyryl-SNAC followed the same method used for the synthesis of Ac-SNAC. The amount of propionic acid used was 200 mg (2.70 mmol), and butyric acid was also 200 mg (2.27 mmol).

### Hydrolytic activity of AlpS.

The reaction system (100 μl) consisted of potassium phosphate buffer (50 mM, pH 7.5), Ac-SNAC (100 μM), and AlpS (2 μM). The reaction was carried out at 37°C and quenched by adding HCl (5 μl, 6 M). The mixture was then extracted with ethyl acetate (100 μl, 2 times), and the organic phase was dried by evaporation. The products was dissolved in DMSO (30 μl) and analyzed by HPLC. The hydrolysis product of propionyl-SNAC reacts with DTNB and form 5-thio-2-nitrobenzoate, which was detected using a microplate reader (maximum wavelength [λ_max_], 412 nm; ε, 13,600 M/cm). The assay mixture (150 μl) contained phosphate buffer (50 mM, pH 7.5), FscTE or AlpS (2 μM), propionyl-SNAC (10 mM), and 10 μl of 10 mM DTNB, and the reaction was carried out on 96-well plates at 37°C for 30 min. The rates of hydrolysis were calculated from the reaction curves from which the rate of the control without enzyme was deducted.

10.1128/mBio.01334-20.4 FIG S4 UV-visible spectra of dehydrorabelomycin (A), Ac-SNAC (B), kinamycin D (C). Download FIG S4, TIF file, 0.5 MB. Copyright © 2020 Hua et al. 2020 Hua et al. This content is distributed under the terms of the Creative Commons Attribution 4.0 International license.
