# Supplementary figures and images for: Offloading Role of a Discrete Thioesterase in Type II Polyketide Biosynthesis
Source: mBio. 2020 Sep 15;11(5):e01334-20. doi: 10.1128/mBio.01334-20 (PMC7492732; doi:10.1128/mBio.01334-20)

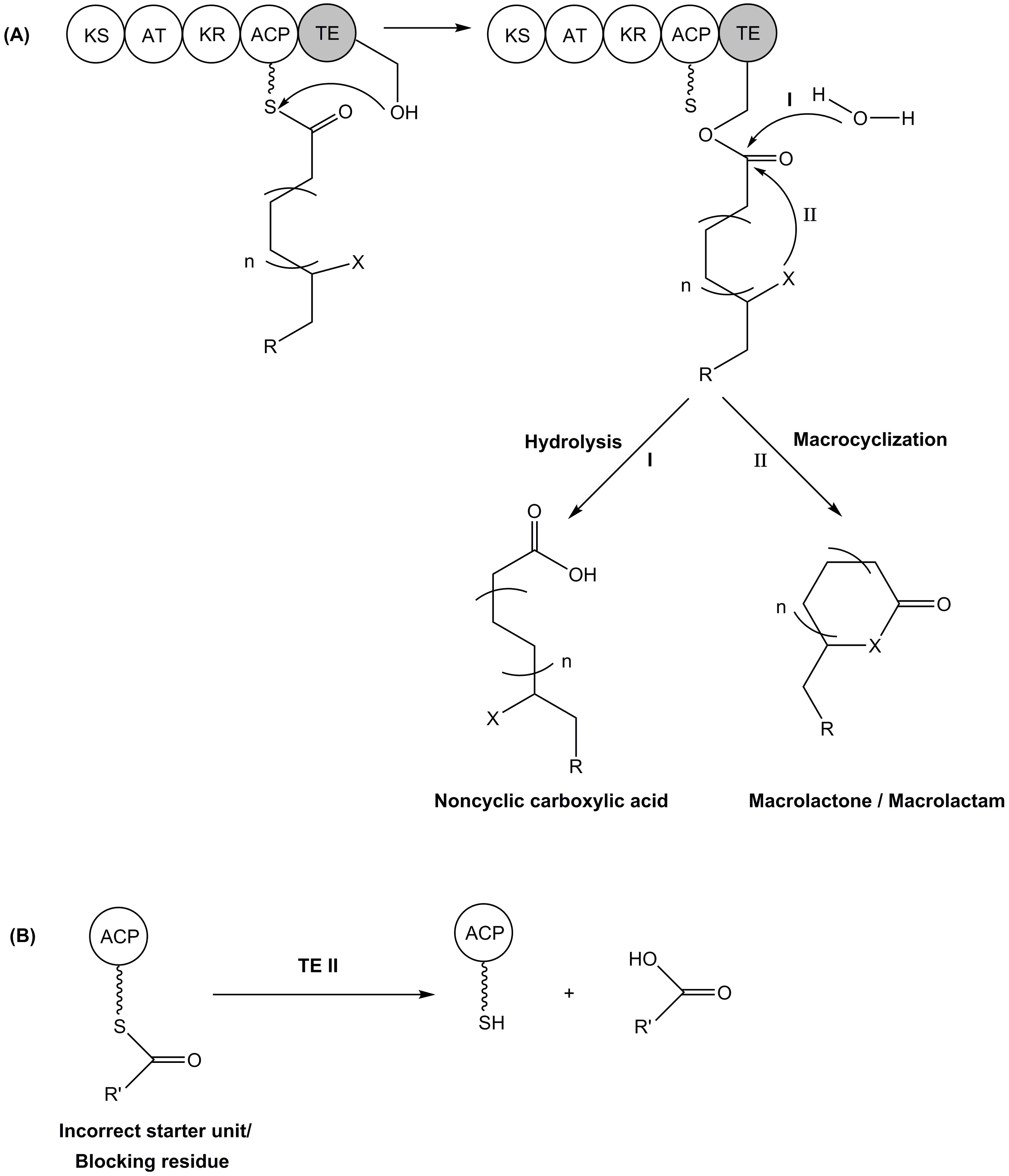

Supplement: FIG S1 [file mBio.01334-20-sf001.tif]

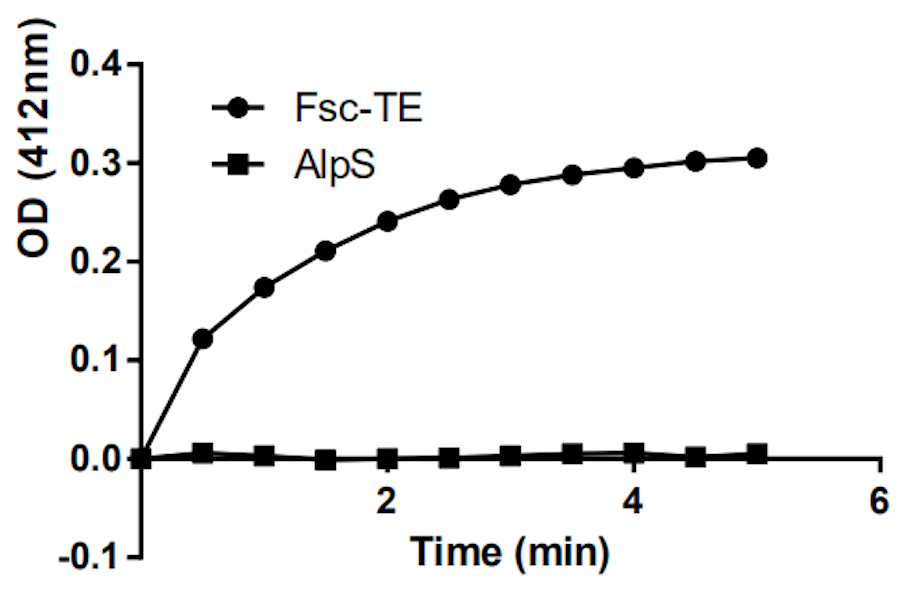

Supplement: FIG S2 [file mBio.01334-20-sf002.tif]

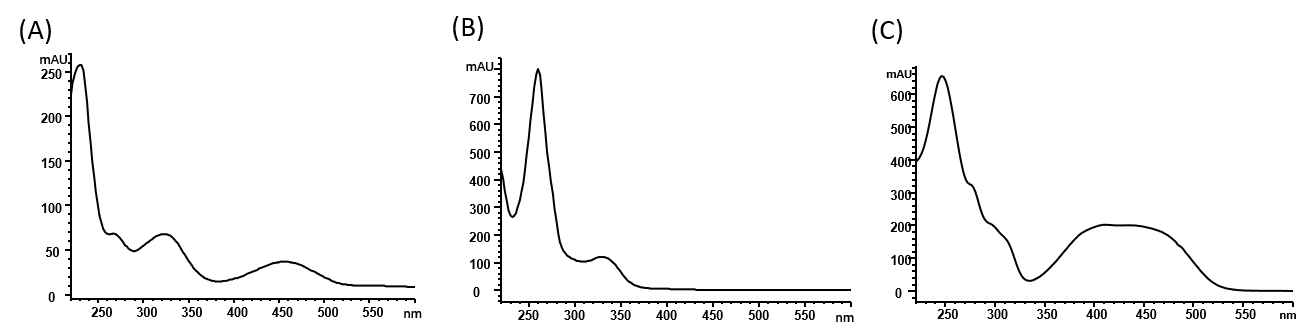

Supplement: FIG S3 [file mBio.01334-20-sf003.tif]

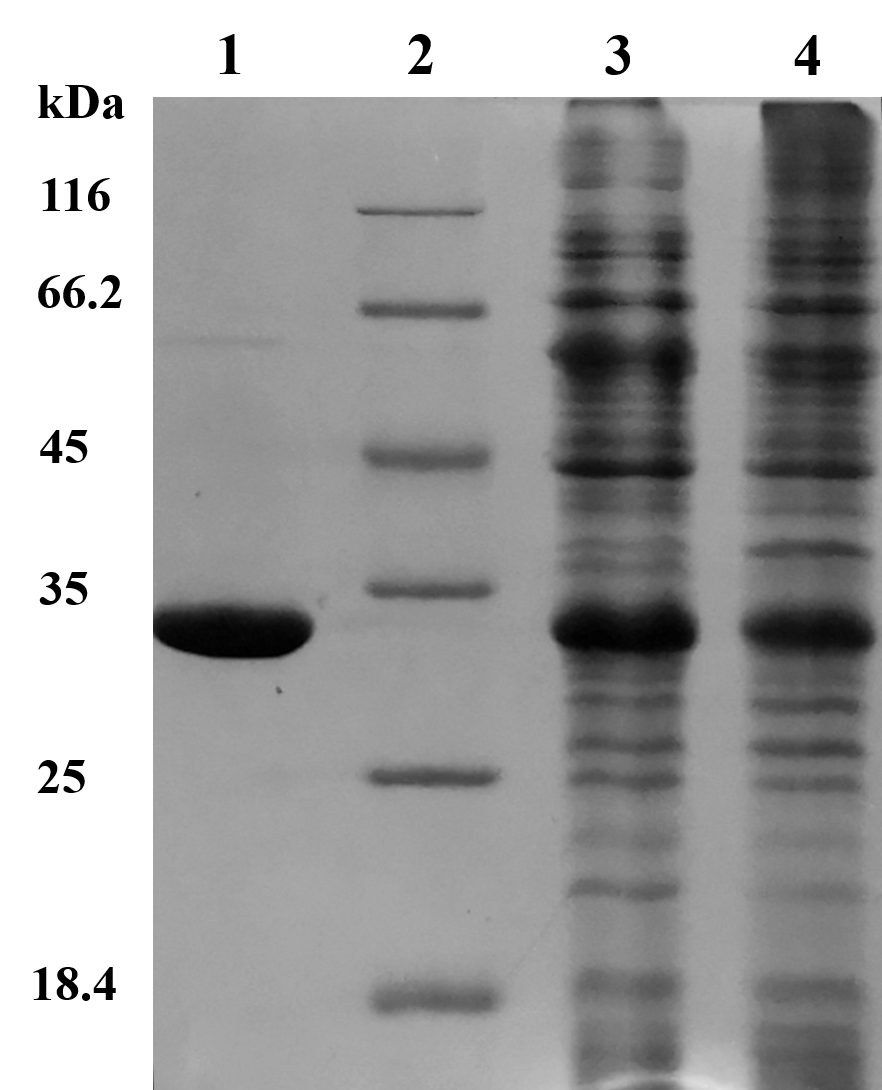

Supplement: FIG S4 [file mBio.01334-20-sf004.tif]
