# Supplementary material for: Offloading Role of a Discrete Thioesterase in Type II Polyketide Biosynthesis
Source: mBio. 2020 Sep 15;11(5):e01334-20. doi: 10.1128/mBio.01334-20 (PMC7492732; doi:10.1128/mBio.01334-20)
Supplement: TABLE S1 [file mBio.01334-20-st001.docx]

| **Strain or Plasmid** | **Relevant Properties** | **Source or Reference** |
| --- | --- | --- |
| ***E.coli*** |  |  |
| DH10B | General cloning and plasmid maintenance | GibcoBRL |
| BL21(DE3)/pGro7 | BL21(DE3) containing plasmid pGro7 | TAKARA |
| BL21/pGro7/pET28a-*alpS* | Expression strain of AlpS | This study |
| BL21/pGro7/pET28a-*alpS(S89A)* | Expression strain of AlpS(S89A) | This study |
| BL21/pGro7/pET28a-*alpS(D202N)* | Expression strain of AlpS(D202N) | This study |
| BL21/pGro7/pET28a-*alpS(H230A)* | Expression strain of AlpS(H230A) | This study |
| BW25113 | K-12 derivative: ∆*araBAD*, ∆*rhaBAD* | ^[1]^ |
| BW25113/2E9-∆*alpW* | BW25113 containing BAC (2E9-∆*alpW*) | ^[2]^ |
| ET12567/pUB307 | *dam dcm hsdS*/pUB307 | ^[3,4]^ |
| ET12567/pUZ8002 | *dam dcm hsdS*/pUZ8002 | ^[4,5]^ |
| BAP1 | Used as the host for heterologous expression | ^[6]^ |
| BAP1/pGro7/pXY-2/pXY-3/pXY-6 | BAP1 containing pGro7, pXY-2, pXY-3, pXY-6 | ^[7]^ |
| BAP1/pGro7/pKM-1/pXY-3/pXY-6 | BAP1 containing pGro7, pKM-1, pXY-3, pXY-6 | This study |
| ***Streptomyces spp.*** |  |  |
| *S. albus* J1074 | Used as the host for heterologous expression | ^[8]^ |
| *S. albus* J1074/2E9*-*∆*alpW* | BAC plasmid pLXY004 integrated into the chromosome of *S. albus* J1074 | ^[2]^ |
| *S. albus* J1074/2E9*-*∆*alpW*∆*alpS* | BAC plasmid pHKM003 integrated into the chromosome of *S. albus* J1074 | This study |
| *S. albus* J1074/2E9*-*∆*alpW*∆*alpS*:: *alpS* | BAC pHKM003 and plasmid pPM927-kasOp**-alpS* integrated into the chromosome of *S. albus* J1074 | This study |
| *S. albus* J1074/2E9*-*∆*alpW*∆*alpS*:: *alpS(S89A)* | BAC pHKM003 and plasmid pPM927-kasOp**-alpS(S89A)* integrated into the chromosome of *S. albus* J1074 | This study |
| **Plasmids** |  |  |
| pGro7 | GroES- GroEL *ori* Cm^r^ | TAKARA |
| pET28a | T7 *lac*, pBR322 origin, Kan^r^ | Novagen |
| pET32a | T7 *lac*，pBR322 origin, Amp^r^ | Novagen |
| pCDF-duet | CDF ori, Str^r^ | Novagen |
| pKM-1 | pET28a-*alpAB*-*MCAT-alpS* | This study |
| pXY-2 | pET28a-*alpAB*-*MCAT* | ^[7]^ |
| pXY-3 | pCDFduet-*alpI*-*ravC* | ^[7]^ |
| pXY-6 | pET32a-*alpD*-*alpE*-*alpF*-*alpG* | ^[7]^ |
| pET28a-*alpS* | pET-28a(+) carrying *alpS* | This study |
| pET28a-*alpS(S89A)* | pET-28a(+) carrying *alpS(S89A)* | This study |
| pET28a- *alpS(D202N)* | pET-28a(+) carrying *alpS(D202N)* | This study |
| pET28a- *alpS(H230A)* | pET-28a(+) carrying *alpS(H230A)* | This study |
| pLXY004 | 2E9-∆*alpW* | ^[2]^ |
| pHKM003 | 2E9*-*∆*alpW*∆*alpS* | This study |
| pJTU968 | Cloning vector, Amp^r^ | This laboratory |
| pJTU968-kasOp* | Modified version of pJTU968 in which *ermEp** is replaced by *kasOp** | This study |
| pJTU968-kasOp**-alpS* | *kasOp** controlled *alpS* in pJTU968 | This study |
| pPM927 | Cloning vector, Str^r^ | ^[9]^ |
| pPM927-kasOp**-alpS* | *alpS* in pPM927 under the control of *kasOp** | This study |

**References**

1.Datsenko, K. A. & Wanner, B. L. One-step inactivation of chromosomal genes in Escherichia coli K-12 using PCR products. Proc Natl Acad Sci U S A. 97, 6640–6645 (2000).

2.Liu, X., Liu, D., Xu, M. et al. Reconstitution of kinamycin biosynthesis within the heterologous host Streptomyces albus J1074. J Nat Prod. 81, 72–77 (2018).

3.Flett, F.,Mersinias, V. & Smith, C. P. High efficiency intergeneric conjugal transfer of plasmid DNA from Escherichia coli to methyl DNA-restricting streptomycetes. FEMS Microbiol Lett. 155, 223–229 (1997).

4.MacNeil, D. J., Gewain, K. M., Ruby, C. L. et al. Analysis of Streptomyces avermitilis genes required for avermectin biosynthesis utilizing a novel integration vector. Gene. 111, 61–68 (1992).

5.Paget, M. S., Chamberlin, L., Atrih, A. et al. Evidence that the extracytoplasmic function sigma factor sigmaE is required for normal cell wall structure in Streptomyces coelicolor A3(2). J Bacteriol. 181, 204–211 (1999).

6.Pfeifer, B. A., Admiraal, S. J., Gramajo, H. et al. Biosynthesis of complex polyketides in a metabolically engineered strain of E. coli. Science. 291, 1790–1792 (2001).

7.Liu, X., Hua, K., Liu, D., Jiang, M. et al. Heterologous biosynthesis of type II polyketide products using E. coli. ACS Chem Biol. 16, (2019).

8.Chater, K. F. & Wilde, L. C. Streptomyces albus G mutants defective in the SalGI restriction-modification system. J Gen Microbiol. 116, 323–334 (1980).

9.Smokvina, T., Mazodier, P., Boccard, F. et al. Construction of a series of pSAM2-based integrative vectors for use in actinomycetes. Gene. 94, 53–59 (1990).
