# Supplementary material for: Offloading Role of a Discrete Thioesterase in Type II Polyketide Biosynthesis
Source: mBio. 2020 Sep 15;11(5):e01334-20. doi: 10.1128/mBio.01334-20 (PMC7492732; doi:10.1128/mBio.01334-20)
Supplement: TABLE S2 [file mBio.01334-20-st002.doc]

| **Primer** | **Sequence (5' to 3')** |
| --- | --- |
| S.gt-∆*alpS*-F | ATGGCTTCCCGCTCCAGGGACCGTGAGGCGGGCACCGCAATTCCGGGGATCCGTCGACC |
| S.gt-∆*alpS*-R | TCACCGGCCGCCTCCCGTGGGCAGCGCGGTCACGTGGCCTGTAGGCTGGAGCTGCTTC |
| S.gt-*alpS*-flank-F | ACCGGACCGTTCACAAGGAG |
| S.gt-*alpS*-flank-R | GACGGTGAGCAGGTCCCAGAA |
| *alpS*-*NdeI*-F | AAACATATGGCTTCCCGCTCCAGGGA |
| *alpS*-*HindIII-SpeI*-R | AAAAAGCTTACTAGTTCACCGGCCGCCTCCCGT |
| *alpS*-*EcoRI*-R | AAAGAATTCTCACCGGCCGCCTCCCGT |
| AlpS-(S89A)-F | GCGCTCTACGGACACGCCATGGGAG |
| AlpS-(S89A)-R | CCGACGAGGGGCCCCATGCTGTGTC |
| AlpS-(D202N)-F | TTCGCGGGCCGCGACAACCCGCTCG |
| AlpS-(D202N)-R | GCGGGTGCGGCGAGCGGGTTGTCGC |
| AlpS-(H230A)-F | ACCGTCGCCGGGGGAGCCTTCTTCG |
| AlpS-(H230A)-R | GAGGAGCTTGCGAAGAAGGCTCCCC |
