# Supplementary material for: Offloading Role of a Discrete Thioesterase in Type II Polyketide Biosynthesis
Source: mBio. 2020 Sep 15;11(5):e01334-20. doi: 10.1128/mBio.01334-20 (PMC7492732; doi:10.1128/mBio.01334-20)
Supplement: TABLE S3 [file mBio.01334-20-st003.doc]

| **Thioesterase** | **Type of TE** | **Protein ID** |
| --- | --- | --- |
| AlpS | Kinamycin-TEII | CAI78382.1 |
| MtmZ | Mithramycin-TEII | CAK50771.1 |
| ORF33 | X26-TEII | AEM44310.1 |
| GrhD | Griseorhodin-TEII | AAM33656.1 |
| Lcz34 | Lactonamycin-TEII | ABX71151.1 |
| San2 | A-74528-TEII | ADG86309.1 |
| SimC2 | Simocyclinone-TEII-1 | AEU17900.1 |
| SimC3 | Simocyclinone-TEII-2 | AAK06795.1 |
| SauT | Aurachin-TEII | ARM20259.1 |
| EncL | Enterocin-TEII | AIN46698.1 |
| ZhuC | R1128-TEII | AAG30190.1 |
| DpsD | Daunorubicin-TEII | AAA65209.1 |
| OxyP | Oxytetracycline-TEII | AAZ78339.1 |
| TylO | Tylactone-TEII | WP_043472398.1 |
| FscTE | Candicidin-TEII | AAQ82559.1 |
| RifR | Rifamycin-TEII | AAG52991.1 |
| PikAV | Pikromycin-TEII | AAC69333.1 |
| EryTII | Erythromycin-TEII | AAU93793.1 |
| GrsT | Gramicidin S-TEII | AEI41826.1 |
| TycF | Tyrocidine-TEII | AAC45933.1 |
| BacT | Bacillorin-TEII | AAD21211.1 |
| SrfD | Surfactin-TEII | AF233756 |
| YbtT | Yersiniabactin-TEII | AAC69590.1 |
| Lch_TE | Lichenysin-TEI | CAA06325 |
| Bat_TE | Bacitracin-TEI | O68008 |
| Srf_TE | Surfactin-TEI | 1JMK_C |
| HctF_TE | Hectochlorin-TEI | AAY42398.1 |
| CrpD_TE | Cryptophycin-TEI | ABM21572.1 |
| DEBS_TE | Erythromycin-TEI | X62569 |
| PICS_TE | Pikromycin-TEI | AF079138 |
| NysK_TE | Nystatin-TEI | AVX51100.1 |
| Ampho_TE | Amphotericin B-TEI | AF357202 |
| Spn_TE | Spinosyn-TEI | AAG23262 |
| MonCII | Monensin-TEII | AF440781 |
| NanE | Nanchangmycin-TEII | AF521085 |
| NigCII | Nigericin-TEII | DQ354110 |
